# Increasing autophagy with fasting and diet: separating fact from fiction

**DOI:** 10.1042/BST20260632

**Published:** 2026-10-13

**Authors:** Sanjna Singh, Timothy J. Sargeant

**Affiliations:** 1 School of Pharmacy and Biomedical Science, College of Health, Adelaide University, Adelaide, Australia; 2 Lysosomal Health in Ageing, Lifelong Health Theme, South Australian Health and Medical Research Institute, Adelaide, Australia

**Keywords:** autophagy, calorie restriction, fasting, intermittent fasting

## Abstract

Autophagy is the cell’s recycling system; it removes unwanted or dysfunctional cellular material, thereby preventing damage. Importantly, it is a key pro-survival pathway during periods of stress, for instance, during nutrient scarcity. As it can support nutrition during starvation, it is not surprising that autophagy is controlled by nutrient availability. As a result, diet-based strategies to modify autophagy have attracted considerable scientific and public interest, although what is presented to the public is often not supported by a solid scientific foundation. Evidence from animal models is compelling: several of these interventions robustly activate autophagy and are linked to improved lifespan and healthspan. Data from humans, however, are much more limited. Many human studies rely on measurement of steady-state levels of autophagy genes or proteins, metrics that do not independently establish pathway activity. Far fewer have measured autophagic ‘flux’, the more rigorous readout of turnover, and where they have, findings are often negative or inconclusive. Here, we review the human research on popular nutritional interventions purported to change autophagy—including prolonged fasting, intermittent fasting, calorie restriction, and altered macronutrient intake—and contrast these findings with the preclinical evidence that has shaped public expectations. We highlight key gaps in the literature, particularly the scarcity of flux-based measurements and the reliance on blood as a proxy for autophagy in other, less accessible tissues. Refining how we monitor autophagy in humans should be a key priority for the field and is ultimately needed to establish whether and how diet modulates human autophagy.

## Introduction

Biological ageing is driven by the deterioration of physiological processes over time, such as (but not limited to) mitochondrial function and proteostasis, and by changes that lead to the accumulation of senescent cells and chronic inflammation [1]; this results in a state of heightened cellular stress. Autophagy—‘*self-eating*’—is a key stress-response pathway that packages and delivers intracellular waste to the lysosome for disposal, maintaining homeostasis. This serves two important functions: (i) by removing cargo such as damaged organelles and protein aggregates, inflammatory signalling, and other age-related damage is suppressed [2]; and (ii) the breakdown and recycling of unwanted material in the lysosome allows cells access to nutrients during times of nutritional stress [3].

Autophagy is a crucial pro-survival mechanism during food scarcity: in animal models, ablating autophagy results in the inability to survive a prolonged fast [4]. Accordingly, nutrient availability is a major regulator of this pathway. Autophagy extends lifespan [5], ameliorates age-related disease [2], and slows down biological ageing [6] in model organisms. Consequently, there is immense interest in using diet to stimulate autophagy and improve human health. However, in certain contexts, autophagy can be harmful. For example, it can support cancer growth by degrading damaged mitochondria [7], providing chemotherapy resistance [8], and enabling immune system evasion [9].

Most of what we know about autophagy and nutrition comes from studies in preclinical models. Work in humans is much more limited; very few claims about diet and autophagy have ever been meaningfully tested in people. Despite this, public interest in dietary strategies purported to boost autophagy has grown rapidly, often outpacing the available scientific literature. Mainstream media outlets and health information sources report on how interventions such as water-only fasting [10], calorie restriction (CR), intermittent fasting (IF), and high-fat, low-carb diets [11] can activate autophagy. More reputable platforms often temper these claims with important caveats, noting both the potential risks of fasting and the limited evidence available in humans. However, this balanced commentary is drowned out by more egregious claims on popular social media platforms such as X, YouTube, Instagram, Facebook, and TikTok, which attract millions of interactions. These include authoritative-sounding but unproven statements such as ‘when you do a dry fast, which means no food no water, your body goes into much deeper autophagy… three times more than a water fast’ [12]. Some sources even acknowledge that they ‘can’t find the research’ but nevertheless claim that autophagy peaks at 48–72 h during a fast [13]. While such content is presented with a degree of certainty that is unsupported by the available evidence, often based on direct extrapolation from animal studies, it is important to note that the proposed biological effects remain plausible; they simply have not been adequately tested in humans. For better or worse, the relationship between autophagy and fasting is a fascinating example of how biochemistry has spilled into the public imagination.

Here, we provide a grounded summary of the science behind autophagy induction, examining the evidence for different nutritional interventions reported to impact autophagy and linking preclinical data to human studies (summarised in Table 1).

**Table 1:** Human autophagy intervention studies

Intervention	*n*	Mean age (years)	Mean BMI (kg/m^2^)	Health status	Duration and details	Tissue	Readout	Result	Limitations	Ref
Prolonged fasting	4; mixed sex (autophagy markers)	43	26	Metabolic disease	72 h	CD45^+^ leukocytes	Protein abundance	↑ LC3-II in 3/4 samples; mixed results for other proteins	Small sample size; some BMI data missing	[14]
Prolonged fasting	Untrained 7; Trained 6; male	Untrained 27; Trained 28	Untrained 26; Trained 24	Healthy	36 h	Skeletal muscle	Protein abundance; protein phosphorylation	↓ pULK1^Ser757^ in trained group; ↓ LC3A/B-I, II and SQSTM1 in untrained group; bidirectional BECN1 changes	Small sample size; no flux-based assessment	[15]
Prolonged fasting	8; male	26	24	Healthy	72 h	Skeletal muscle	Protein abundance; protein phosphorylation	↓ p-ULK1^Ser757^, ↑ LC3B-II/I and SQSTM1	Single sex; small sample size; no flux-based assessment; LC3B-II/I ratio used	[16]
Prolonged fasting	Lean 9; Obese 9; male	Lean 24; Obese 24	Lean 21; Obese 36	Normal weight or obese; otherwise healthy	72 h	Skeletal muscle	Transcript abundance; protein abundance; protein phosphorylation	↑ ULK1 and SQSTM1 transcripts; ↑ ULK1 protein; ↑ SQSTM1 protein (lean only); ↓ p-ULK1^Ser757^, LC3B unchanged	Single sex; small sample size; no flux-based assessment; LC3B-II/I ratio used	[17]
Prolonged fasting	11; mixed sex (autophagy markers)	56	26	Healthy	26 h	Adipose tissue	Transcript abundance	↑ GABARAPL1; no change in other autophagy-related transcripts	Small sample size; no flux-based assessment	[18]
Prolonged fasting	9; mixed sex	25–54 (mean unavailable)	20–25 (mean unavailable)	Healthy	24 or 48 h (autophagy markers)	Leukocytes	Protein abundance; autophagic flux	24 h fast: *ex vivo* ↑ LC3B-II, no change in flux; 48 h fast: LC3B-II and SQSTM1 unchanged	Small sample size; use of cell culture media for *ex vivo* measurements	[19]
Fasting-mimicking diet (FMD)	Control 9; ProLon 11; FMD2 10; mixed sex	Control 45; ProLon 52; FMD2 49	Control 25; ProLon 29; FMD2 31	Overweight to obese; otherwise healthy	5 days; FMD groups: 1100 kcal day 1, 700–800 kcal/day days 2–5	PBMCs	LC3B-II/I ratio ± lysosomal inhibitor	No change in LC3B ‘flux’ (as defined by authors); reported ↑ from baseline with ProLon but derivation unclear	Baseline BMI differences between groups; LC3B-II/I ratio used to calculate ‘flux’	[20]
Intermittent fasting (IF)	IF70 17; IF100 14; female	IF70 49; IF100 51	IF70 31; IF100 32	Obese; otherwise healthy	8 weeks; 24 h-fast 3 days/week; 70% (IF70) or 100% (IF100) baseline energy needs	Skeletal muscle	Transcript abundance	↓ BECN1 with IF70; ↓ SQSTM1 with IF70 and IF100 (compared with baseline, measured on a fed day)	No flux-based assessment	[21]
Time-restricted eating	11; mixed sex	32	30	Obese; otherwise healthy	4 days; energy-balanced; 12 h versus 6 h eating window	Blood	Transcript abundance	↑ LC3A morning after day 4	Small sample size; no flux-based assessment	[22]
Intermittent time-restricted eating (iTRE) and calorie restriction (CR)	Control 23; CR 44; iTRE 47; mixed sex	Control 61; CR 58; iTRE 57	Control 34; CR 35; iTRE 35	Obese; at risk of diabetes; otherwise healthy	6 months; CR: 70% baseline energy needs; iTRE: 30% before noon + 20-h fast 3 days/week	PBMCs	Autophagic flux	↑ LC3B-II flux at 6 months (iTRE versus control); no significant within-group change from baseline	Single tissue; no cell-type resolution	[23]
CR	Control 10; CR 15 (transcript cohort); Control 10; CR 14 (protein cohort); mixed sex	59 (transcript cohort)	Control 25; CR 19	Healthy	6 years (mean); ∼30% CR	Skeletal muscle	Transcript abundance; protein abundance	↑ ULK1, ATG101, BECN1, LC3, ATG16L1, ATG4B and GABARAPL2 transcripts; ↓ ATG12 transcripts; ↑ LC3B and BECN1 protein	No flux-based assessment	[24]
CR	Control 33; CR 57	Control 40; CR 38	Control 25; CR 25	Healthy	2 years; 12% CR	Skeletal muscle	Transcript abundance	↑ ATG13 with CR	No flux-based assessment	[25]
CR	38; mixed sex	46	45	Severely obese	24 weeks; 6 weeks at 654 kcal/ day then 18 weeks at 1200–1800 kcal/day	PBMCs	Protein abundance	↑ LC3A/B-II/I and NBR1 post-intervention	LC3A/B-II/I ratio used; no flux-based assessment	[26]
Macronutrient composition—protein intake	Study 1: 12; Study 2: 7; mixed sex	Study 1: 41; Study 2: 41	Study 1: 28; Study 2: 28	Healthy	Single meal after overnight fast; Study 1: 10% versus 50% protein; Study 2: 15% versus 22% protein	Monocytes	LC3 immuno - fluorescence	↓ LC3 1 h and 3 h after high versus low protein meal (Study 1); ↓ LC3 2 h after a higher versus lower protein meal (Study 2)	No flux-based assessment	[27]
Macronutrient composition—protein intake	40; mixed sex	33	25	Healthy	Single high-protein drink after overnight fast	PBMCs	Autophagic flux	No change in LC3B-II flux 1 h after meal	Single postprandial time-point	[28]
Macronutrient composition—protein restriction	7; mixed sex	32	23	COL6- related myopathies	12 months; ≤0.68 g protein/kg /day	Skeletal muscle	Transcript abundance; protein abundance; transmission electron microscopy	↑ LC3B (7/7) and BECN1 (6/7) transcripts; ↑ LC3B-II (7/7) and BECN1 (6/7) protein; ↑ autophagic vesicles (7/7)	Small sample size and disease status; no flux-based assessment	[29]
Macronutrient composition—FMD with protein manipulation	Control 16; FMD-LP 15; FMD-HP 15; mixed sex	Control 35; FMD-LP 38; FMD-HP 41	Control 23; FMD-LP 23; FMD-HP 25	Healthy	7 days; 850 kcal/day; FMD-LP 10% protein; FMD-HP 30% protein	Blood	Transcript abundance (whole blood); protein abundance (plasma)	↑ LC3A and ATG4B transcripts with FMD-LP; ↑ ULK1 and BECN1 transcripts with FMD-HP; ↑ GABARAPL1 and ATG2A transcripts with both diets; no change in autophagy-related proteins measured	No flux-based assessment	[30]
Macronutrient composition—protein restriction	61; mixed sex	29	25	Healthy	4 weeks per diet; energy-balanced; 20% versus 10% protein	PBMCs	Autophagic flux	No change in LC3B-II flux	Single tissue; overnight-fasted measurements only; no cell-type resolution	[31]
Macronutrient composition—protein restriction	19; mixed sex	LP 47; HP 49	LP 46; HP 44	Severely obese	3 weeks; ∼1500 kcal/day with 10% versus 30% protein	Liver	Transcript abundance; autophagic flux	↑ LC3B-II flux in LP versus HP group; ↑ LC3A and ATG5 transcripts with LP	Small sample size; differences in baseline hepatic fat between groups; flux not significant after adjustment for baseline hepatic fat	[32]
Macronutrient composition—dietary fat	39; mixed sex	HSFA 58; HMUFA 57; LFHCC 57; LFHCC n-3 55	HSFA 36; HMUFA 35; LFHCC 36; LFHCC n-3 35	Metabolic syndrome	12 weeks; HSFA, HMUFA, LFHCC or LFHCC n-3 diets	Adipose tissue	Transcript abundance	↑ BECN1 and ATG7 from baseline with HMUFA	Small sample size; no flux-based assessment	[33]

Abbreviations: BMI, body mass index; CR, calorie restriction; FMD, fasting-mimicking diet; HP, high-protein; LP, low-protein; IF, intermittent fasting; PBMC, peripheral blood mononuclear cells; HMUFA, high-monounsaturated fatty acid diet; HSFA, high-saturated fatty acid diet; LFHCC, low-fat high-complex carbohydrate diet; LFHCC n-3, low-fat high-complex carbohydrate diet supplemented with n-3 polyunsaturated fatty acids.

## Molecular links between nutrient availability and autophagy

Mechanistically, autophagy is a multi-step process that typically begins at ER-associated sites, whereby a double-membraned structure called the phagophore is formed under the control of the ULK1 complex (comprising ULK1 and 2, ATG13, ATG101, and RB1CC1/FIP200) [34–36] and the class III phosphatidylinositol 3-kinase (PI3KC3) complex I (comprising PI3KC3/VPS34, BECN1, PIK3R4/VPS15, and ATG14) [37,38]. As the phagophore expands, ATG8 proteins, most prominently, the widely used autophagy marker MAP1LC3B/LC3B, are covalently attached to phosphatidylethanolamine on the growing phagophore membrane via two ubiquitin-like conjugation systems, forming lipidated LC3B-II. Once sufficiently expanded, this structure seals to form a double-membraned organelle termed an autophagosome. Cargo enters this pathway either through bulk non-selective engulfment of cytosolic contents or through interactions (direct or indirect) with ATG8 proteins on the expanding phagophore. Eventually, autophagosomes mature and fuse with lysosomes, where the acidic, hydrolase-rich environment degrades the inner autophagosomal membrane and its contents.

This pathway receives input from the cell’s two major energy sensors: mechanistic target of rapamycin kinase complex 1 (mTORC1) and AMP-activated protein kinase (AMPK). AMPK is activated when cellular energy is low, whereby it facilitates the initiation of autophagy by phosphorylating ULK1 [39,40] and by suppressing mTORC1 activity [41,42]. mTORC1 acts as a coincidence detector that receives and integrates information from two nutrient-relevant streams: amino acid availability and insulin and growth factor signalling. It localises to the lysosome in response to amino acid detection, where it is activated by the small GTPase RHEB in response to insulin and growth factor receptor signalling through tuberous sclerosis complex proteins [43]. In its active form, mTORC1 is the primary negative regulator of autophagy [44], via suppressive phosphorylation of multiple proteins along the autophagy pathway, including ULK1 and 2 [35,36,40] and ATG14 [45]. mTORC1 also suppresses the transcription of autophagy and lysosome proteins through phosphorylation of MiT/TFE family transcription factors, including TFEB, TFE3, and MITF [46–48]. Thus, autophagic activity is closely coupled to nutrient availability: nutrient scarcity inhibits mTORC1 and stimulates autophagy, whereas nutrient abundance activates mTORC1 and suppresses autophagy.

## Measuring autophagy: methods, challenges, and pitfalls

Measurement of autophagy is inherently challenging [49], with numerous approaches used across the literature [50]. However, not all methods are created equal; different readouts carry different ‘weights’—taking this into consideration is crucial when interpreting the evidence. Many studies employ steady-state measurement of autophagy-related transcripts or proteins. Although such readouts can show that, for example, ULK1 or BECN1 has been up-regulated, they do not indicate autophagic activity, as expression and functional turnover do not necessarily correlate [51]. This is even true for the canonical autophagy marker lipidated LC3B, where an increase in abundance could indicate either increased induction or impaired degradation, ultimately providing very little information on actual pathway activity. Further, while LC3B-II is present on autophagosomes, it can also be conjugated onto other organelles in an autophagy-independent manner, often termed conjugation of ATG8s to single membranes, or CASM (for instance, onto endo-lysosomal membranes in response to sphingomyelin exposure [52,53]). In addition, LC3B-II is often quantified relative to its unlipidated form (LC3B-II/I ratio) rather than a loading control—something that is explicitly advised against in the autophagy community guidelines, as antibody affinity differs between the two forms and LC3B-I abundance can vary independently of LC3B-II [50].

Caution must also be exercised when assessing the abundance of the receptor sequestosome 1 (SQSTM1/P62), another frequently used autophagy readout. SQSTM1 binds autophagic cargo and is itself degraded in the lysosome; thus, lower SQSTM1 levels are often interpreted as an increase in autophagy. However, transcriptional programmes can very quickly increase expression of SQSTM1 during stimuli such as nutrient restriction [54] and oxidative stress via NRF2 [55] and TFEB [56], which confounds interpretation and severely limits the utility of this measurement.

To reliably gauge autophagy, flux-based metrics, which capture the movement of material through the autophagy-lysosome pathway over time, are recommended [50]. Fluorescent reporters can exploit the change in pH that accompanies delivery of autophagic material to the acidic lysosomal environment. For example, the tandem fluorescent RFP-GFP-LC3B probe shifts from yellow to red as acid-sensitive GFP fluorescence is quenched [57], while mKeima-based reporters undergo a pH-dependent shift in excitation [58]. Such tools provide a functional readout of autophagic trafficking to the lysosome. Variations on this concept (including ATG4-cleavable GFP-LC3-RFP-LC3ΔG [59]) have improved measurement techniques and expanded their utility [60]. While extremely useful, these markers are of course restricted to systems that can be genetically modified, such as cells and animal models.

Given that such techniques cannot be applied to primary human samples, monitoring human autophagy necessitates the use of lysosomal inhibitors to measure turnover. Here, living cells (in fresh whole blood, for instance) are split into two parallel samples, one of which receives a lysosomal inhibitor. Incubation with the lysosomal inhibitor allows autophagic material to accumulate over time, with the difference between the two samples representing the amount of cargo that would otherwise have been degraded over this period, i.e. autophagic flux. Applying this approach to human studies requires careful consideration. Preserving the physiological environment is particularly important, as isolating primary cells or tissue explants removes them from circulating nutrients, hormones, and other systemic signals that may influence autophagy. Blood is therefore an attractive and readily accessible tissue, as flux can be measured in whole blood [61], largely preserving the extracellular milieu. Indeed, isolation of peripheral blood mononuclear cells (PBMCs) prior to measurement can dramatically reduce autophagic flux [62].

However, measurement of autophagy in whole blood has caveats—for example, if chloroquine is used as the lysosome inhibitor, CASM could make up a proportion of the signal. Further, the blood sample is no longer *in vivo*, and autophagic activity may be influenced by transition to *ex vivo* conditions. PBMC autophagy may also not be representative of what is occurring elsewhere; in a mouse model of ageing, we have observed that it does not correspond to other tissues such as the heart or brain [63]. Additionally, changes to the composition of the PBMC pool could influence results: we have shown that different subpopulations in the PBMC fraction differ both in baseline autophagy and in the response to amino-acid restriction [62]. This can be resolved by cell-type-specific measurement of autophagic flux or at minimum, monitoring PBMC subpopulations such that the experimenter is at least aware of potential confounds.

## Prolonged fasting to activate autophagy

There is strong evidence from preclinical models that prolonged fasting activates autophagy as a pro-survival mechanism. Seminal work from the Ohsumi lab revealed that nutrient restriction activates the autophagy pathway in budding yeast [64]. Years later, using a transgenic mouse expressing GFP-LC3B to measure autophagosome abundance [65], they showed that 24 or 48 h of fasting profoundly stimulated autophagy across different tissues (excluding the brain, although other evidence suggests that fasting may also stimulate neuronal autophagy [66]). Recently, a more sophisticated reporter corroborated these findings, showing robust activation of autophagy in the liver and muscle, but not brain, of mice that had been fasted for up to 48 h [67]. Consistent with this, autophagy-related transcripts such as LC3B and the cargo adaptor SQSTM1 also increased in various tissues over this timespan [68].

Yet the evidence that prolonged fasting, defined here as the absence of calories for 24 h or longer, stimulates autophagy in humans is limited and inconsistent. Proteomic profiling of human leukocytes after a 3-day fast revealed increased abundance of several autophagy-related proteins such as BECN1 and ATG5; this was accompanied by an increased LC3B-II/I ratio [14]. In human skeletal muscle, 36 h of fasting altered several autophagy-related markers in a training-dependent manner, although autophagic flux was not assessed [15], and a longer 72-h fast caused a decrease in mTORC1 activity and a corresponding increase in the LC3B-II/I ratio [16]. However, in a separate study that also examined skeletal muscle in subjects who had undergone a 72-h fast, LC3B remained unchanged, while transcript and protein levels of other autophagy-related machinery such as SQSTM1 and ULK1 increased [17], though, as noted above, changes in SQSTM1 are ambiguous with regard to autophagic activity. In human adipose tissue, a 26-h fast had little effect on broader autophagy-related gene expression, although expression of GABARAPL1, an ATG8-family protein, increased [18].

Another group reported increased autophagy based on changes to LC3B-II and SQSTM1 levels in circulating leukocytes from human volunteers who had fasted for 24 h [19]. However, there was no concomitant increase in flux, making interpretation difficult. Moreover, this only occurred when leukocytes were isolated from human blood and placed into cell culture for 1 h; if the cell culture step was omitted, even a longer 48-h fast failed to elicit a detectable autophagic response. Another limitation here is that placing cells in cell culture media—which contains supraphysiological levels of glucose and amino acids—dramatically alters their physiological environment, which has itself been shown to reduce autophagic flux [62].

Recently, fasting-mimicking diets (FMD), where caloric intake is markedly reduced, have been proposed as a safer, more feasible alternative to prolonged fasting while still retaining some of its health benefits [69] and are discussed here as a related but distinct intervention. After a 5-day period, FMD increased steady-state LC3B-II/LC3B-I levels relative to baseline, but there was no increase in autophagic flux as measured via an *ex vivo* assay in whole blood [20]. To date, no equivalent flux data exist for prolonged fasting in humans, although a registered trial protocol describes a randomised crossover study that will assess autophagy using this same assay after a 3-day fast in humans [70]. Even so, this approach is not without limitations: PBMC autophagy does not always correspond to turnover in other cell types in mice [63] and humans [71].

Overall, to date there is no definitive human evidence showing if, when, and in which tissues induction of autophagy occurs during a prolonged fast.

## Does meal timing matter? Intermittent fasting and time-restricted feeding/eating

IF is a dietary strategy that involves a period of fasting interspersed with periods of *ad libitum* food consumption. Time-restricted feeding/eating (TRF/TRE) is a type of IF that limits food intake to a defined daily eating window. Importantly, neither approach necessarily involves intentional CR, although in practice reductions in energy intake often occur. In recent years, IF has gained popularity as a dietary regimen to facilitate weight loss, reduce cardiovascular disease risk, and improve age-associated metabolic defects [72].

There is an emerging body of evidence suggesting that altering meal timing may have an impact on autophagy. In *Drosophila*, 20-h fasting on alternate days induced autophagy via several metrics: first, there was an increase in pro-autophagy signalling, as evidenced by increased AMPK phosphorylation and reduced TOR signalling [73]. Expression of core autophagy genes was enhanced in the IF flies; this was accompanied by an increase in autophagy as measured via a functional autophagic turnover assay. Overall, this iTRF regimen extended *Drosophila* lifespan by 13%–18% in a manner that was autophagy-dependent: flies lacking key autophagy genes did not show increased lifespan.

Previous work has demonstrated that a regimen comprising 24-h fasts on three non-consecutive days per week increased transcript and protein levels of some autophagy markers in mouse liver, but not in mouse muscle; however, given the absence of functional autophagy assessments, it remains unclear whether these changes reflect increased autophagic activity [21]. Measurement of autophagic flux in mice has yielded conflicting results. In mice subjected to an alternate-day fasting IF regimen, autophagy increased during fasting as expected; however, no differences were detected in either autophagosome puncta or autophagic flux in the heart when animals were examined on a fed day [74]. This suggests that autophagy in these mice depended on the nutritional state of the animal, rather than the feeding schedule itself. In contrast, a TRF study—where mice received the same total daily food intake as *ad libitum* controls but were restricted to two 2-h daily feeding windows—reported the opposite conclusion [75]. Under these conditions, autophagic flux increased markedly in the liver immediately after feeding, despite concurrent activation of mTORC1 signalling. Similar postprandial increases in autophagic flux were observed in brown adipose tissue, skeletal muscle, and the hypothalamus [75], suggesting that this response occurs across multiple tissues. The simultaneous postprandial activation of mTORC1 and autophagy diverges from the canonical model in which nutrient intake suppresses autophagy through activation of mTORC1 signalling [35,40], suggesting that under specific physiological conditions, autophagy may become uncoupled from mTORC1 regulation.

Although the effects of meal timing on autophagy remain unclear, several lines of evidence suggest that autophagy contributes to at least some of the physiological benefits associated with IF interventions. In the TRF mouse study, tissue-specific deletion of the essential autophagy gene *Atg7* attenuated several metabolic adaptations to the dietary regimen [75]. Similarly, alternate-day fasting reduced myocardial ischaemia–reperfusion injury in wild-type mice, but not in *Lamp2* heterozygous mice, which have impaired autophagic turnover [74]. Importantly, in *Lamp2* heterozygous mice, IF actually worsened the autophagic flux defect [74], suggesting that IF may not be a suitable dietary approach in all scenarios and requires a suitable level of autophagy–lysosome pathway capacity in order to exert some of its cardioprotective effects. Together, these preclinical findings suggest that while the extent and timing of autophagy induction may vary between protocols, functional autophagy appears to be required for at least some of the benefits attributed to IF.

In humans, very few studies have specifically examined the relationship between IF and autophagy. In one report, a TRE schedule that involved a daily 6-h feeding window increased transcript abundance of autophagy-related genes like MAP1LC3A/LC3A (a paralogue of LC3B) in blood [22]. Another study found increased transcript levels of ATG5 and ULK1, but a decrease in BECN1, in blood over the course of a month-long religious fast comprising a daily 17–19 h fasting window [76]. In human muscle, there was a decrease in BECN1 transcripts with a 24-h fasting on three non-consecutive days per week IF regimen [21]. While these studies show that IF can alter autophagy-related machinery, they did not establish whether autophagic activity changed. Recently, we measured autophagic flux in the PBMCs of participants following a modified IF protocol where energy intake was restricted by 70% on three non-consecutive days per week to be consumed during a 4-h feeding window [23]. Compared with standard care, participants assigned to the IF intervention had higher autophagic flux after 6 months. However, within the IF group, autophagic flux did not increase significantly relative to baseline, and the study was not designed to determine whether this effect was sustained beyond 6 months. Further, it remains unclear whether the observed changes in flux were driven by energy restriction, altered meal timing, or both.

Collectively, while preclinical studies suggest that meal timing can influence autophagy, and intact autophagy may contribute to some of the protective effects of IF, human data remain extremely limited; it is unclear whether IF reliably enhances autophagy in humans or whether certain regimens are more effective than others.

## Calorie restriction and autophagy

CR, the reduction of daily caloric intake by 20%–40% without malnutrition, prolongs lifespan in a variety of model organisms, including worms [77], mice [78], and perhaps even non-human primates [79]. Autophagy may contribute to some of these longevity and health-promoting effects: CR shifts the circadian rhythm of mTOR signalling in mice [80], and the mTORC1 inhibitor rapamycin can mimic some of the protective effects of CR (although this is not direct causal evidence, given that mTORC1 signalling also regulates numerous other cellular processes) [81]. Further, when autophagy is inhibited in *Caenorhabditis elegans*, the pro-longevity benefits of CR disappear [82].

However, whether CR induces autophagy in mammals remains unclear. One study showed that 12 months of CR increased both LC3B immunofluorescence and lipidated LC3B abundance, and reduced SQSTM1 protein levels in mouse kidney, indicative of increased autophagy [83]. However, functional pathway turnover was not assessed. In mouse muscle, CR does not appear to increase either steady-state autophagy protein abundance [84] or flux [85]. In the brain, CR increases autophagy protein abundance over time in mice [86]. In contrast, a different study in rhesus monkeys found that CR did not alter levels of several autophagy proteins in the brain as assessed via western blot [87].

Data on long-term CR and autophagy in humans are scarce, likely due to difficulty with adherence. Increases in autophagy-related markers in muscle have been reported, including LC3B, ULK1, and BECN1 transcripts and LC3B and BECN1 protein levels after 3–15 years of CR [24], as well as ATG13 transcripts after 2 years of 12% CR [25]. A shorter, 6-month period of CR increased the LC3B-II/I ratio in PBMCs from individuals with obesity [26]. However, in a recent study where we compared CR to standard care in a cohort at increased risk of type 2 diabetes, over a 6-month period, we were unable to detect any changes to autophagic flux in PBMCs [23]. Differences in participant characteristics, tissue sampled, duration and intensity of the intervention, and autophagy readouts used (steady-state abundance versus flux assay) limit direct comparisons between the studies. Overall, both preclinical and human data are ambiguous with regard to whether CR stimulates autophagy.

## The effect of macronutrient composition on autophagy

Altering macronutrient composition, particularly protein intake, is another strategy that may modulate autophagy, with increased dietary protein expected to suppress this process. This is because mTORC1 receives direct input from amino-acid sensing pathways. Dietary protein and amino acid intake positively correlate with mTORC1 signalling in mice [88,27], rats [89], and humans [27,28], as assessed via phosphorylation of its downstream substrates. In turn, modifying mTORC1 activity regulates autophagy; in rats and mice fed a low-protein diet comprising 10% protein, autophagy protein levels increased in the liver, and in mice this was accompanied by an increase in liver autophagic flux [90]. Similarly, reducing protein intake to 5% of total calories in rats raised protein levels of the autophagy marker LC3B-II in the kidney [89], although this study did not examine flux.

In humans, the effect of dietary protein on autophagy appears more complex. A single high-protein meal elevates circulating amino acids and increases mTORC1 activity in peripheral blood cells [27,28]. However, whereas one study showed a corresponding decrease in LC3B immunofluorescence in monocytes [27], another report measuring LC3B autophagic flux in the bulk PBMC pool found no effect [28]. Although this could be driven by differences in readouts used between the two studies, another possibility is that different immune cell types may simply vary in terms of their autophagic responsiveness to protein intake. In line with this, we have shown that in cultured human PBMCs, complete amino acid restriction increased autophagic flux only in monocytes [62]. Collectively, these findings suggest that autophagic responses to protein modulation may be cell-type specific.

The effect of longer-term protein restriction in humans is unclear. One study reported that reducing protein intake to 0.6 g/kg body weight for 1 year in patients with COL6 myopathies increased LC3B-II and BECN1 protein abundance in muscle and leukocytes [29]. However, the absence of flux measurements, together with the small sample size and underlying disease, makes it difficult to determine whether these findings would translate to healthy individuals. A recent report comparing a standard diet to high- and low-protein versions of an FMD observed only modest transcriptional changes in a small number of autophagy genes, without corresponding changes at the protein level [30]. Similarly, in a randomised crossover study comparing standard- and low-protein intake over 4 weeks, we found no changes in PBMC autophagic flux between the two diets [31]. However, a different study in individuals with morbid obesity reported higher autophagic flux in *ex vivo* liver samples after 3 weeks on a low-protein diet (compared with a high-protein diet), although this difference was no longer significant after adjustment for higher baseline hepatic fat in the low-protein group [32]. Thus, although current evidence does not consistently demonstrate that protein modulation can change autophagic flux in humans, it should be noted that human flux data are limited to three reports, only one of which was powered using prior flux measurements, estimating 53 participants would provide ∼90% power to detect a 15% within-subject effect [31]. As a difference of this magnitude approaches the detection sensitivity of the assay used in that study [23], smaller effects as well as tissue-specific responses cannot be excluded.

Compared with dietary protein, there is little evidence examining the effects of other macronutrients on autophagy. In mice, a high-fat diet increases autophagic flux in white adipose tissue [91] and pancreatic beta cells but reduces liver autophagic flux [92]. Similarly, mice fed a ketogenic diet—a nutritional strategy involving very low-carbohydrate intake coupled with high fat intake—showed increased protein levels of autophagy markers in the brain [93] and increased flux in the liver [94]. Together, these preclinical findings suggest that dietary fat may influence autophagy in a tissue-specific manner. However, whether dietary fat similarly regulates autophagy in humans is unknown, although increased adipose BECN1 and ATG7 transcript levels have been reported after 12 weeks of a monounsaturated fat-rich diet [33].

## Conclusions and future directions

While there is a large body of evidence suggesting that autophagy can be altered through various dietary strategies in animal models, most of these have not yet been well-characterised in humans (Figure 1). Further, seemingly minor differences in dietary protocols can produce markedly different outcomes. For example, IF increased lifespan in *Drosophila* with a 20-h fast every other day, yet reduced lifespan when the fasting period was extended to 24 h [73]. Further, lifespan extension required night-biased fasting. Such findings suggest that the details of a dietary intervention, including its timing, intensity, and duration, may have important consequences for autophagy and health.

**Figure 1:**
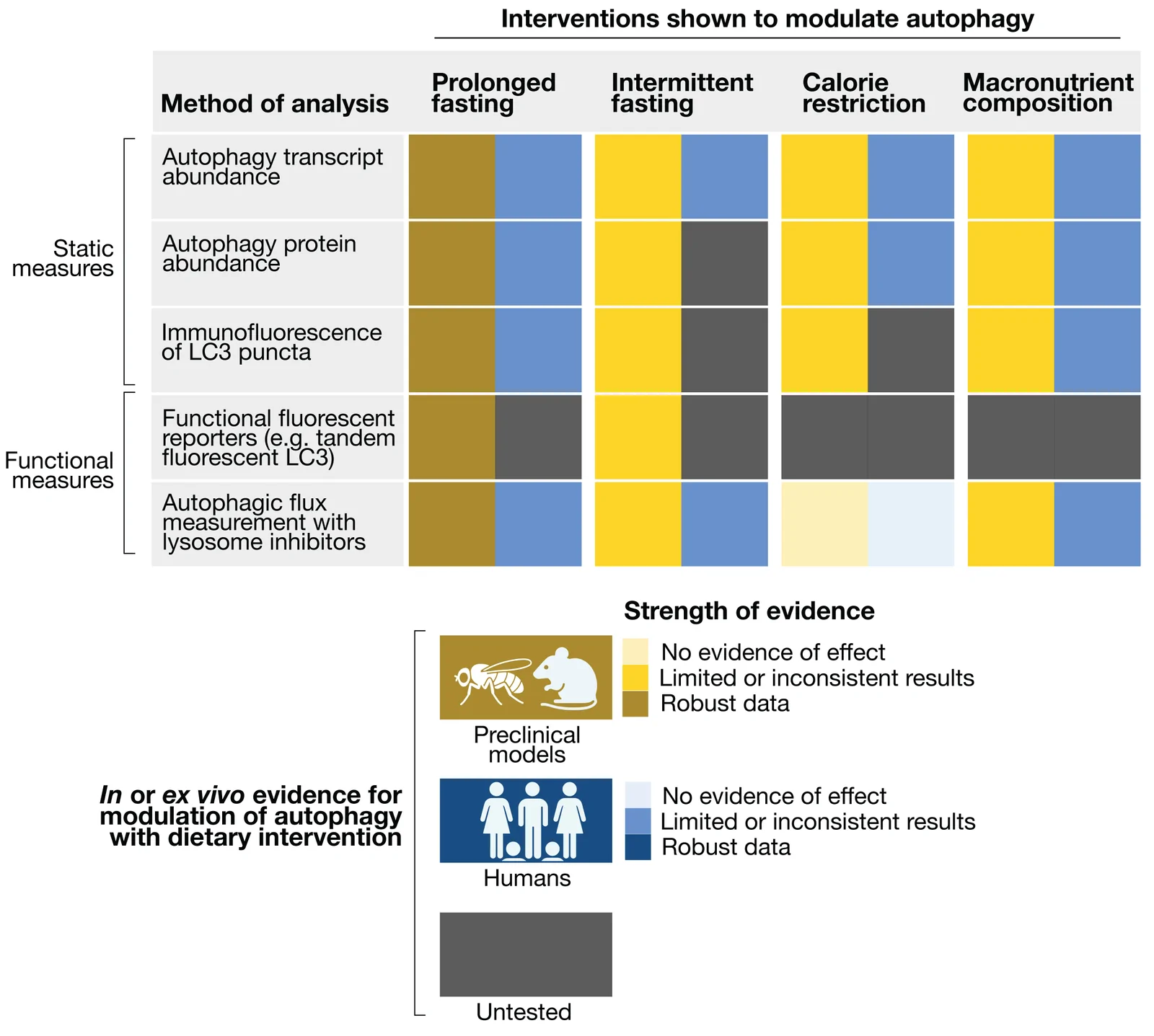
Summary of studies reporting dietary modulation of autophagy Shading indicates the strength of evidence demonstrating an effect for each dietary intervention, considering the consistency of findings within and across studies. Created in BioRender. Sargeant, T. (2026) https://BioRender.com/x1utcnt.

Despite decades of research, at present, our understanding of human autophagy remains remarkably limited. Even fundamental concepts, such as how long a human must fast for in order to activate autophagy, remain unresolved. In the absence of conclusive human data, a significant amount of preclinical research has been treated as directly equivalent. For instance, the common perception that 24–72 h of fasting stimulates autophagy in people likely stems from landmark mouse studies [65]. However, fasting in mice is not comparable to that in humans: mice start to die after 3 days without food [95], whereas humans can tolerate at least 21 days [96], with an extreme example being 382 days [97], albeit under medical supervision with access to vitamins, yeast, minerals, and non-caloric fluids. Human and mouse physiology has diverged greatly in the 90 million years since we shared our last common ancestor [98]. Humans have much lower energy metabolism than mice [99], with approximately eight-fold lower-weight corrected energy expenditure. Mice also deplete their liver glycogen stores more quickly and rely far more heavily on gluconeogenesis: after just 4 h without food, gluconeogenesis accounts for 80% of endogenous glucose production in mice compared with 40%–50% in humans after an overnight fast [100]. Hormone signalling in response to nutrient restriction is also altered. In mice, serum levels of the stress-inducible hormone fibroblast growth factor 21 (FGF21) increase after 6 h of fasting, preceding the rise in ketones at 24 h [101]. In contrast, FGF21 in humans does not peak until 10 days of fasting—7 days after the peak in serum ketones [101]. In summary, the mouse response to fasting is condensed and subject to much higher metabolic demands. The markedly different temporal dynamics of FGF21 and ketogenesis during fasting further illustrate why findings from mice cannot be directly extrapolated to humans.

Given these differences, advancing the field will require rigorous studies in humans to determine which dietary interventions truly modulate autophagy and whether findings from preclinical models translate to meaningful effects on human health. Moreover, this needs to be done in a manner that actually reports autophagic flux, rather than relying on steady-state levels of autophagy-related transcripts or proteins. At present, blood is the only easily accessible human tissue amenable to such assays, and there is a growing body of work investigating the impact of lifestyle interventions on autophagic flux in human PBMCs [71,23,28]. However, we do not yet know how generalisable such measurements are to other tissues. The development and implementation of improved approaches for measuring autophagy in humans will be a key step in determining which interventions alter human autophagy and under what conditions they do so. Priority areas should include, firstly, refining measurement of autophagic flux in humans. Blood is the only tissue that can be removed intact and maintained as its own culture system; however, muscle and adipose biopsies are also routinely taken from study participants. Understanding whether such samples can be used for flux measurements would represent a significant advance. Secondly, the development of biofluid-borne biomarkers, either single molecules (RNA, lipid, protein, or metabolite) or panels of molecules that correlate with autophagy, remains a major unmet goal in the field. Such biomarkers would have a substantial impact on accelerating the clinical development of autophagy-based interventions. However, they would need to be carefully mechanistically linked to autophagy, and their limits—at a minimum, how synthesis, secretion, and elimination interact with autophagy—would need to be well defined. Until then, much of what is claimed about dietary modulation of autophagy in humans remains plausible but ultimately unproven.

## Literature search

For this narrative review, relevant literature was identified through searches of PubMed and Google Scholar using combinations of terms relating to autophagy, fasting, caloric restriction, protein restriction, high-protein and ketogenic diets (e.g. ‘human’ AND ‘fasting’ AND ‘autophagy’; ‘human’ AND ‘fasting’ AND ‘LC3B’). Additional studies were retrieved from the reference lists of relevant articles. Searches included literature published up to September 2026. Relevant human studies identified using this approach are summarised in Table 1.

## Perspectives

- Autophagy is a fundamental, nutrient-sensitive pathway linked to healthy ageing, and dietary strategies to boost it—from fasting to CR—have generated enormous public and scientific interest, often ahead of the evidence needed to support them.
- Although preclinical models show robust autophagy induction in response to various dietary interventions, whether these effects translate to humans remains unclear, reflecting both fundamental physiological differences between species and the difficulty of measuring autophagic activity meaningfully in humans.
- Progress will depend on rigorous, flux-based human studies, but also the development of tissue-specific biomarkers of autophagy. Until these tools exist, claims about diet and human autophagy will remain largely unsubstantiated.

## Open Access

Open access for this article was enabled by the participation of Adelaide University in an all-inclusive Read & Publish agreement with Portland Press and the Biochemical Society under an agreement with CAUL.

## Abbreviations

AMPK: AMP-activated protein kinase
FGF21: fibroblast growth factor 21
FMD: fasting-mimicking diets
IF: intermittent fasting
mTORC1: mechanistic target of rapamycin kinase complex 1
PBMCs: peripheral blood mononuclear cells
PI3KC3: phosphatidylinositol 3-kinase
TRE: time-restricted eating
TRF: time-restricted feeding
CR: calorie restriction
